# An Integrated Framework for the Implementation and Strengthening of Antimicrobial Stewardship Programs in Six Countries in Latin America

**DOI:** 10.3390/antibiotics15050497

**Published:** 2026-05-15

**Authors:** Gabriel Levy-Hara, Paola Lichtenberger, Robin Rojas-Cortes, José Pablo Diaz-Madriz, Pilar Ramon-Pardo, Jose Luis Bustos, Anahi Dreser Mansilla, Tania Herrera, Marisol Cofre, Irene Pagano, Marcela Rojas, Giovanna Huaquipaco, Noemí Lugo, Tatiana Orjuela Rodriguez, Diego Macías Saint-Gerons, Didia Sagastume, Jose Luis Castro, on behalf of the Latin American PPS Group

**Affiliations:** 1 Hospital de Agudos Carlos G. Durand, Buenos Aires 1405, Argentina; 2 Infectious Diseases Division, University of Miami Miller School of Medicine, Miami, FL 33136, USA; paola.lichtenberger@va.gov; 3 Pan American Health Organization, Washington, DC 20037, USA; rojasedg@paho.org (R.R.-C.); ramonpap@paho.org (P.R.-P.); sagastudid@paho.org (D.S.); 4 Hospital Clínica Bíblica, San Jose 10903, Costa Rica; jp.diazm27@gmail.com; 5 Independent Consultant, Guatemala City 01015, Guatemala; jluis859@gmail.com; 6 National Institute of Public Health, Cuernavaca 62100, Mexico; anahi.dreser@insp.mx; 7 Ministry of Health, Santiago 8320064, Chile; tshm17@hotmail.com (T.H.); marisol.cofre@minsal.cl (M.C.); 8 National Institute of Epidemiology, Mar del Plata 7600, Argentina; irenepagano4@gmail.com; 9 Ministry of Health and Social Protection, Bogota 111711, Colombia; mrojas@minsalud.gov.co; 10 General Directorate of Medicines, Supplies and Drugs, Lima 15088, Peru; ghuaquipaco@minsa.gob.pe; 11 Federal Commission for Protection Against Health Risks, Mexico City 03810, Mexico; dgplades.noemi@gmail.com; 12 Independent Consultant, Bogota 20037, Colombia; ltorjuelar@unal.edu.co; 13 Faculty of Nursery, Universidad de Valladolid, 47002 Valladolid, Spain; maciasdie@paho.org; 14 Independent Consultant, Buenos Aires 1058, Argentina; castrojl@paho.org

**Keywords:** antimicrobial stewardship programs, Latin America, implementation

## Abstract

Background: Antibiotic overuse in hospitals is common and linked to adverse outcomes and antimicrobial resistance. Antimicrobial stewardship programs (ASP) aim to optimize prescribing and require context-specific adaptation. Objectives: To describe the experience of implementing and strengthening ASP in hospitals from six Latin American countries by using an integrated framework. Methods: The intervention included a point-prevalence survey (PPS) of antibiotic use, a baseline checklist, a continuous online education program, and individual facility meetings to share SWOT analyses and recommendations. The latter was performed based on PPS and checklist results. The checklist covers six domains (authorities’ commitment, organization, structure, and accountability; interventions; education and training; monitoring and surveillance; and internal communication). The training program spanned 12–18 months and addressed core ASP components. Results: The PPS across 67 hospitals showed an antibiotic use prevalence of 47.9%, with 63% of prescriptions deemed appropriate. The median checklist score was 61.2%. Among the categories assessed, monitoring and surveillance achieved the highest score (median 75.0; IQR 63.9–84.0), while education received the lowest (median 43.8; IQR 29.7–62.5). A total of 80 country groups and 35 individual hospital meetings were held. Conclusions: An integrated, data-driven framework combining PPS, checklists, individual hospital meetings, and sustained training provides a scalable approach to strengthening ASP in diverse Latin American hospitals, aligning with Pan American Health Organization (PAHO) guidance and global recommendations.

## 1. Introduction

In hospital settings, antibiotics are among the most frequently prescribed medications. However, studies have shown that up to 50% of antibiotic use is inappropriate or unnecessary, causing an economic burden and patient harm in terms of poor treatment outcomes, adverse events, and increased antimicrobial resistance (AMR), a major global health threat associated with increased mortality and longer hospitalization [1,2,3,4].

Antimicrobial stewardship programs (ASPs) are critical components of modern healthcare systems, particularly in hospitals where the misuse and overuse of antibiotics pose significant challenges [5,6]. These programs aim to improve the use of antimicrobials and the quality of patient care by reducing antimicrobial resistance (AMR), healthcare-associated infections, and healthcare costs, among other objectives [5,6,7,8]. The interventions derived from the ASP aim to ensure that prescribers use the right drug, dose, and route during the treatment period.

The World Health Organization (WHO) and the Pan American Health Organization (PAHO) emphasize that ASPs must be tailored to the specific context and resources of each institution. To this end, they recommend conducting a situational assessment that includes identifying the existing basic components and available resources required to establish an ASP, as well as analyzing the main challenges related to antimicrobial prescribing practices within the hospital. In this regard, developing a point-prevalence survey (PPS) and a baseline checklist can be a useful starting step.

A PPS is a cross-sectional audit of antimicrobial prescribing practices conducted at a single point in time, providing critical data on patterns of antibiotic use, appropriateness, and resistance [3]. The checklist, which includes the various components of an ASP (e.g., commitment of authorities, organization, interventions for optimizing antimicrobial use), allows for the assessment of the program’s status—both at baseline and over time—and identifies its main weaknesses, enabling the team to design strategies to gradually improve it in line with its capabilities.

By integrating data from tools such as PPS’s and checklists, ASP teams can move from general assumptions to an evidence-based SWOT (Strengths, Weaknesses, Opportunities, and Threats) analysis, enabling them to prioritize effective, local interventions in line with global recommendations [5,6].

Between 2018 and 2019, a PPS of antibiotic use was conducted across 33 hospitals in five Latin American countries to assess antimicrobial use and prescribing practices [9]. The survey revealed widespread issues, including high rates of inappropriate antimicrobial prescribing, excessive use of broad-spectrum agents, and prolonged antimicrobial treatments. Most hospitals faced significant challenges after obtaining these results, given that the COVID-19 pandemic exacerbated longstanding problems such as scarcity of the necessary tools, resources, and expertise to analyze the data and identify specific weaknesses within their ASP. As a result, hospitals struggled to develop context-specific action plans to improve antimicrobial prescribing. This situation highlighted a critical gap not only in data analysis capacity but also in the ability to link PPS results to practical, institution-specific stewardship strategies, ultimately limiting the potential impact of the survey on antimicrobial use and patient outcomes.

In September 2021, PAHO developed an integrated framework to strengthen ASP in hospitals across six Latin American countries. This initiative provided hospitals with structured support to conduct a checklist and a PPS, and to perform a SWOT analysis and formulate a data-driven action plan for those facilities that completed both tools. Additionally, this holistic intervention included a continuous, structured online educational program to provide the ASP teams with the required tools to effectively translate survey findings into sustainable, context-adapted improvements in antimicrobial prescribing practices.

The aim of this manuscript is to describe the experience of implementing and strengthening ASPs by using integrated tools and training interventions in hospitals from six countries in Latin America. Since the PPS results have recently been published [10], we include only the key findings to highlight their importance within the framework of an integrated project.

## 2. Methodology

The project, performed between 2022 and 2024, included six countries and was conducted with the support of PAHO and regional experts through a strategic three-step approach consisting of: (1) baseline assessment using PPS and PAHO checklists, (2) expert-led SWOT analysis to identify gaps, followed by the development of tailored annual action plans, and (3) continuous training on implementation of ASP.

The selection of hospitals was carried out jointly by PAHO country offices and officials from different branches of the Ministries of Health (MoH) in each country. A local research team was assembled and received training and supervision to apply the tools. A summary of the methodologies used is provided below.

### 2.1. Baseline Assessment Using PPS on Antibiotic Use in Hospitals and PAHO Checklist

#### 2.1.1. Baseline PPS Assessment

The Latin American PPS methodology has been previously published [10] and consists of a slightly modified Spanish translation of the WHO PPS methodology [3]. Among the countries involved in this project, only Argentina did not participate in the PPS because its hospitals regularly conduct this study in the context of the National Program of Infection and Prevention Control (VIHDA).

#### 2.1.2. Baseline Checklist of Antimicrobial Stewardship Programs

The checklist was developed based on existing tools produced by different organizations [5,8,11] and validated using a modified Delphi technique with Latin American experts. The tool allows for systematic evaluation of the main components and actions of the ASP, both at baseline and periodically (e.g., annually), facilitating the assessment of their progress and determining priorities for improvement. It consists of a self-assessment survey organized in 77 questions distributed among six sections: commitment of the authorities/directors; organization, structure, and accountability; interventions aimed at optimizing the use of antimicrobials; education and training; monitoring and surveillance; and communication within the health facility. Each question has five possible answers, and each answer has a numerical correspondent score ranging from 0 to 100: fully implemented (100), partially implemented (75), planned but not initiated (50), not planned but considered a priority (25), and not considered for implementation (0).

The analytical approach varied depending on the specific variable. For the six ASP components, medians and interquartile ranges (IQR, 25th–75th percentiles) were used due to the asymmetric nature and high dispersion of the data. In turn, means were used for the analysis of main variables within each component, given that—as medians represent the most chosen value—the conditions answered as “not implemented” would jeopardize a more realistic picture of the current situation. Since hospitals were selected using different criteria and the sample was heterogeneous, it is not suitable to perform a reliable statistical analysis to compare the levels of development of the components.

### 2.2. Expert-Led SWOT Analysis to Identify Gaps, Integrating Analysis of PPS and Checklist Results

Only institutions that completed both the PPS and the structural assessment checklist were invited to participate in individualized, stakeholder-driven meetings. These criteria were based on the consideration that only by combining the results of both baseline studies would it be possible to perform an adequate analysis to formulate recommendations. The interdisciplinary approach—comprising hospital authorities, unit heads, ASP leaders, clinicians, and Infection Prevention and Control (IPC) members—was designed to facilitate a comprehensive analysis of local situations and to generate tailored, actionable recommendations.

The coordinating team analyzed PPS and checklist results to produce an integrated SWOT analysis and specific recommendations for improving their program. The analysis mainly focused on optimizing the top three prescribed antimicrobials and empiric therapy for the top three most common diagnoses. The findings highlighted organizational and structural opportunities for improvement and were presented to each facility in an individual meeting. After discussing the SWOT results and recommendations, each local team developed a prioritized annual action plan incorporating two or three targeted strategies.

### 2.3. Data Management and Storage

Both the PPS and the checklist were designed and implemented through a web-based platform built on REDCap (Research Electronic Data Capture). REDCap is a secure, web-based electronic data capture application designed for research data management, developed by the Vanderbilt Institute for Clinical and Translational Research at Vanderbilt University (Nashville, TN, USA) [12].

To ensure data confidentiality and integrity, the project was hosted on an isolated server environment. A secure communication protocol based on Secure Sockets Layer (SSL) encryption was implemented to protect data transmission. In addition, access to the project was restricted to authorized collaborators through individualized login credentials (username and password), thereby reinforcing data security and controlled access. Data access permission was restricted to the coordination team and hospital team coordinators. To ensure comprehensive, multidisciplinary input and minimize bias, the hospital coordinators were advised to complete both surveys jointly with their ASP teams.

## 3. Results

### 3.1. Baseline Assessment Using PPS on Antibiotic Use in Hospitals and PAHO Checklist

#### 3.1.1. Point Prevalence Survey on Antibiotic Use in Hospitals

Since the results of the Latin-PPS 2022–2023 used for the integrated analysis were recently published [10], only the key findings that help to understand the relevance of this survey within the overall intervention are summarized here.

The study was conducted between March 2022 and November 2023 and included 67 hospitals from five countries: Chile (*n* = 13), Colombia (*n* = 8), Mexico (*n* = 22), Panama (*n* = 1), and Peru (*n* = 23). Main results showed that 5310 out of 11,094 patients surveyed (47.9%) were receiving at least one antibiotic, with considerable variations among countries: Chile (39.0%), Colombia (46.0%), Peru (51.7%), Mexico (53.4%), and the Panamanian hospital 59.1% [10].

Ceftriaxone (17.9%), meropenem (9.3%), vancomycin (9.2%), and metronidazole (7.6%) were the frequently prescribed antibiotics. According to WHO’s AWaRe classification (11), most prescriptions corresponded to the Watch group (58.2%), followed by Access (38.5%) and Reserve (3.2%).

Sixty-three percent of prescriptions were considered compliant with CPGs, showing higher adherence in Chile and Colombia (~75%). Compliance was considerably higher for treatment (70%) than for prophylaxis (30%). In the latter, surgical prophylaxis showed an adherence rate of only 22%, mainly due to prolonged administration (more than 24 h) in 62% of cases [10].

#### 3.1.2. Baseline Checklist of Antimicrobial Stewardship Programs

**Figure 1:**
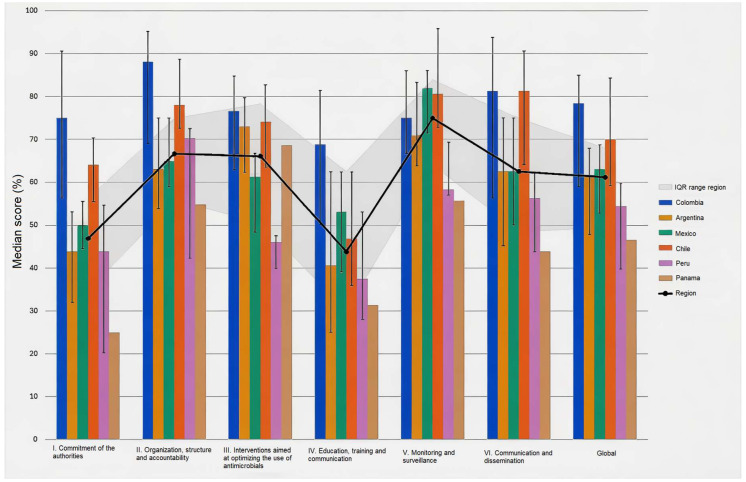
Bar chart of baseline checklist PAHO/WHO results, 2022–2023.

Between August 2022 and March 2024, 121 hospitals from six countries completed the baseline self-assessment of their ASP with a median score of 61.2% (Table 1 and Figure 1).

Colombian hospitals (*n* = 49) showed a higher score of ASP implementation level (median, 78.4; IQR, 59.0–85.0), followed by Chile (*n* = 8, median; 70.0; IQR, 59.2–84.3), Mexico (*n* = 18; median, 63.1; IQR, 52.8–68.8), Argentina (*n* = 38; median, 61.2; IQR, 47.8–68.0), Peru (*n* = 7; median, 54.4; IQR, 39.7–59.8) and Panama (*n* = 1; median, 46.5).

The components that displayed the highest scores were monitoring and surveillance (median, 75.0; IQR, 63.9–84.0), organization, structure, and accountability (median, 66.7; IQR, 55.7–75), and interventions aimed at optimizing the use of antimicrobials (median, 66.1; IQR, 50.6–78.4). On the other hand, commitment of the authorities (median, 46.9; IQR, 34.4–56.3) and education and training (median, 43.8; IQR, 29.7–62.5) were the lowest-ranked.

While statistical analysis of the data was not performed due to heterogeneity of the sample, notable trends were observed (Table 1 and Figure 1). Colombia showed a good performance in all components; Chile scored high overall, except in the area of education; Argentina showed good progress regarding interventions for optimizing antimicrobial use and monitoring, but poor performance in terms of support from authorities and education; Mexico showed strength in monitoring and surveillance but reported lower levels of support from authorities and education; and Peru reported good levels of organization and structure of ASPs, with low levels of commitment from authorities, education, and interventions for optimizing antimicrobial use.

### 3.2. Analysis of Each Component of the ASP

The results of each component are shown in Table 2. As described in the Section 2, and to reflect them as accurately as possible, selected variables are presented below, showing cumulative results (expressed in means), and including only positive responses (i.e., fully and partially implemented).

#### 3.2.1. Commitment of Hospital Authorities

In 61.9% of facilities, the optimization of antimicrobial use was considered a priority by authorities, and in 62% of cases, the ASP was formalized. On the other hand, only 22.3% of them received specific funding (Table 2a).

#### 3.2.2. Organization, Structure, and Accountability

Table 2a shows that almost all hospitals have a clinical microbiological laboratory (CML) and the majority receive samples daily and perform cultures with sensitivity tests. Seventy-one percent have a leader specialized in ASP; 64.4% reported the existence of an AMS Committee; 52.1% allocated some protected time for the leaders of the program, more frequently to the infectious diseases specialist (57.8%) than for the pharmacist (43%). In 45.4% of cases, other health-care workers (HCWs)—outside the ASP team—were involved in antimicrobial optimization activities.

#### 3.2.3. Interventions to Optimize Antimicrobial Use

Pre-authorization for prescribing certain antimicrobials (77.6%), switch to oral route (75.2%), review of prescriptions within 48–72 h (73.5%) and the use of some clinical practice guidelines (67%) were the prevalent tools reported, followed by joint clinical rounds with prescribers (62.9%) and the requirement to fully document the indication for antimicrobials in clinical records (62%). In turn, dose adjustments for organ failure (48%) and monitoring drug levels (e.g., vancomycin) (38.8%) were less commonly reported (Table 2b).

#### 3.2.4. Education and Training

Table 2c shows that while 52.9% of hospitals trained ASP team members on AMS and IPC, and that 57.9% provided continuous staff education, only 36.3% of hospitals offered initial education to staff, and 34.7% education to patients and their relatives.

#### 3.2.5. Monitoring and Surveillance

Prevalent activities consisted of monitoring multidrug-resistant (MDR) infection rates (95%), followed by monitoring shortages of essential antimicrobials (82.7%), and producing cumulative antibiograms of key bacteria (73.5%). Antimicrobial use or consumption studies (57%) and the use of AWaRe classification (35.6%) were less frequently reported (Table 2c).

#### 3.2.6. Internal Communication

In 60.3% of hospitals, the antimicrobial susceptibility of prevalent bacteria was analyzed and shared with prescribers, and in 51.2%, data on acquisition, prescription, and dispensing were disseminated. In turn, 43% of facilities communicated the results of audits or reviews of the quality and appropriateness of antimicrobial use to prescribers, accompanied by specific action recommendations (Table 2c).

### 3.3. Integrated Analysis of PPS and Checklist Results

From July 2023 to March 2024, 35 individualized meetings were held to provide feedback on the analysis of the integrated results of the PPS, the baseline self-assessment, the SWOT analysis, and the recommendations. Fourteen meetings were held with Mexican hospitals, nine with Chilean hospitals, six with Peruvian hospitals, five with Colombian hospitals, and one with the Panamanian facility.

A trend was observed between the PPS results and the checklist results. Countries with higher levels of ASP development (i.e., Chile, 70.0%; Colombia, 78.4%) showed lower prevalence of antibiotic use (39.0% and 46.0%, respectively) than those with lower ASP development (i.e., Peru, 54.4%; Mexico, 63.1%), which presented higher prevalence rates of antibiotic use (51.7% and 53.4%, respectively). However, due to the limited number of observations (*n* = 4), no inferential statistical analysis of correlation was performed.

Regardless of these trends, most hospitals faced essentially similar issues. Therefore, despite some differences related to the degree of development of each hospital program, key aspects of the SWOT analysis were common (Table 3).

In view of this SWOT analysis, recommendations provided to hospital ASP teams generally included considerations related to administrative, organizational, and educational aspects, as well as interventions aimed at optimizing the use of antimicrobials and microbiological diagnosis (Table 4).

### 3.4. Continuous Training on Implementation of ASP

From January 2022 to December 2023, regular meetings were established with participating countries. Participation of countries’ representatives ranged from 20 for Chile to 85 for Peru.

A Google Drive folder of each country was shared with participants, containing the project documentation. This repository included CPGs, key research papers, documents essential for the PPS (such as the protocol in Spanish, REDCap security information, and an ethical committee submission model), presentations (both PPT files and links to meeting recordings), and meeting minutes. Since new hospitals were integrated throughout the process, several key aspects of AMS were revisited periodically. This approach strategically paired hospital ASP teams with significant experience with those just beginning.

By the conclusion of the training program, a total of 35 meetings were held with hospitals in Chile, 14 in Argentina, 12 in Mexico, 11 in Peru, and 8 in Colombia. Additionally, in-person workshops were conducted in Mexico and Chile.

## 4. Discussion

Since the early 2000s, and with increasing momentum over the past decade, ASP has been adopted across Latin American hospitals; however, progress remains heterogeneous, constrained by region-specific barriers [13,14,15,16].

Checklist results confirm that structural and cultural limitations, such as restricted protected time and limited access to informatics tools, hinder implementation of an effective ASP [14,15,17,18]. Although the baseline assessment showed that nearly two-thirds of participating programs have formalized administrative structures and policies, these efforts remain insufficient to overcome identified operational obstacles. While 61% of institutions reported administrative support, merely 22% secured dedicated financial backing. Even though some protected time is allocated for 58% of ID specialists and 43% of pharmacists, the actual requirements to improve ASP performance are fulfilled in only 32% and 16%, respectively. Although these figures are higher than recent findings from Mexico—where only 12% of ASP leaders have protected time (22% private vs. 6% public) [15]—they remain inadequate. Without formal time allocation, stewardship activities are treated as supplementary tasks, contributing to staff burnout and neglecting critical intervention efforts.

Another major obstacle is that fewer than half of the hospitals involved professionals not on the ASP team, partially due to poor internal communication regarding the program’s activities and results. Given the limited core human resources in most ASP teams, disseminating the program through dedicated focal points in all inpatient units should be crucial for its success.

While checklists revealed that many hospitals possess well-equipped CML operating extended hours, both country-level sessions and individual hospital integration meetings highlighted a significant underutilization of these resources. This finding is consistent with the results of our first PPS study [9], in which samples for microbiological testing were collected in only 44% of cases, 82% of antibiotic treatments were empirical, and merely 17% of treatments were adjusted based on laboratory results. Consequently, a frequent recommendation was to maximize CML utilization—specifically through improved sampling, timely reporting, and treatment de-escalation—alongside the routine production of cumulative antibiograms.

On the other hand, the good news is that most facilities implemented a wide range of interventions to optimize antimicrobial use (e.g., pre-authorization and/or supervision of use through audits and feedback, joint rounds with prescribers, 72 h reviews). However, advanced technical strategies, including therapeutic drug monitoring and adjusted dosing for organ injury, remain less frequently adopted (38% and 48%).

Data from the PPS and the checklist suggest a trend in which countries with more advanced ASPs—such as Chile and Colombia—have a lower prevalence of antibiotic use and greater adherence to CGP guidelines, while Peru—with less developed programs—showed higher utilization rates. Mexico deviated from this trend: despite an intermediate level of ASP development, it recorded the highest prevalence of antibiotic use (excluding Panama due to the aforementioned limitations). The relationship between higher checklist scores and lower antibiotic consumption aligns with previous literature [19].

There are many possible explanations for this apparent inverse trend between program developments and antibiotic use. One of the main reasons might be that primary efforts appear focused on consumption surveillance, leaving insufficient time for supervising antimicrobial use, training, stakeholder engagement, and internal communication. Among other things, this hinders the adoption of surgical prophylaxis guidelines, particularly given surgeons’ frequent resistance to changing their practices. Another important driver is limited internal communication, which prevents physicians from understanding the importance of the program and the implications of not adhering to interventions aimed at improving the quality of antimicrobial use.

The combined analysis of PPS and checklist results allowed us to address these systemic hurdles by fostering a culture of objective evaluation. It emerged that the weaknesses identified (e.g., funding, technological) were, to a large extent, offset by the ASP team’s commitment. The presentation and discussion of this analysis and our recommendations during hospital individualized meetings helped institutions to translate raw performance data into actionable insights, enabling the development of a tailored, feasible annual plan focused on high-impact, low-cost interventions (e.g., prospective audit, feedback on specific high-cost wards, monitoring selected CPGs). Furthermore, since one of the recommendations was to present the results of the analysis to the authorities and prescribers, this was—on several occasions—the first step toward improving internal communication within the program.

On the other hand, country group meetings provided an opportunity to train ASP teams in a practical way by applying evidence-based strategies for low- and middle-resource hospitals. Participants acquired competencies in aligning institutional policies with national resolutions, incorporating ASP into the hospital’s organizational structure, and implementing strategies to obtain protected time and funding.

This intervention has several limitations. First, hospital selection criteria were different across participating countries. In Colombia, Chile, and Panama, facilities with prior experience in ASP were included, whereas institutions from Peru, Mexico, and Argentina had heterogeneous levels of ASP development. This variability likely influenced both the checklist and the PPS results. Second, the wide variation in the number of hospitals included limits the possibilities of extrapolating the results to a national level. Third, the PPS consists of a self-reporting study conducted at a specific point in time, which may be affected by modifying factors—such as seasonality or outbreaks of MDR bacteria, and therefore does not reflect trends over time. Lastly, the checklist has inherent risks associated with self-reporting, such as the overestimation of various program components. To minimize reporting biases as much as possible, there were oversight procedures in place. In the case of the PPS, the data uploaded to the REDCap were daily checked, and inconsistencies were discussed with the local study coordinators. Regarding the checklist, the information was reviewed during individual meetings with the hospitals, and the data initially provided was generally found to be accurate. Although these meetings were held with only 35 of 121 (29%) hospitals, during the country group sessions, most hospitals reported challenges, and program development levels aligned with the checklist results.

A key strength of this study is its comprehensive, integrated approach. It enabled many hospitals across different countries to take part in training and discussion sessions and share their experiences—both positive and negative—with their peers. The SWOT analysis, followed by specific recommendations, provided ASP teams with essential tools to produce their annual action plans tailored to each hospital’s specific context. By combining quantitative data from the PPS with qualitative structural evaluations, this multidimensional framework provides a more comprehensive view of stewardship capacity.

Once the phase of this project presented here was completed, the coordinating team continued to support countries both through group meetings and individual contacts with hospitals that had specific requests. During the second half of 2025, part of the project was resumed involving Argentina, Chile, Colombia, Mexico, Peru, and Paraguay. The intervention included country group meetings, a new round of checklists, and individual hospital meetings, without performing a PPS. It was noted that most hospitals had continued to develop their ASP, and that new facilities joined the initiative. The analysis of this new experience is currently underway and will be the subject of a future publication.

Essentially, it is likely that the main factor leading to the long-term success of these comprehensive interventions lies in the support of governments and the definition of clear and binding national policies, enabling facilities to implement and subsequently sustain programs and—ideally—improve their standards year after year. Ideally, this support should be sustained over time regardless of changes in government and health authorities, as it is a priority public health policy. Continued collaboration between MoHs and governing organizations has proven essential for enhancing ASP.

## Figures and Tables

**Table 1:** General results of baseline checklist, PAHO/WHO, 2022–2023.

	Region	Colombia	Argentina	México	Chile	Perú
	Median (IQR 25–75)	Median (IQR 25–75)	Median (IQR 25–75)	Median (IQR 25–75)	Median (IQR 25–75)	Median (IQR 25–75)
I. Commitment of the authorities	46.9 (34.4–56.3)	75.0 (56.3–90.6)	43.8 (32.0–53.1)	50.0 (44.5–55.5)	64.1 (55.5–70.3)	43.8 (20.3–54.7)
II. Organization, structure, and accountability	66.7 (55.7–75.0)	88.1 (69.1–95.2)	63.1 (53.9–75.0)	64.9 (58.9–75.0)	78.0 (72.6–88.7)	70.2 (42.3–72.6)
III. Interventions aimed at optimizing the use of antimicrobials	66.1 (50.6–78.4)	76.6 (62.9–84.7)	73.0 (62.3–79.8)	61.3 (48.4–66.7)	74.2 (63.5–82.7)	46.0 (39.9–47.6)
IV. Education and training	43.8 (29.7–62.5)	68.8 (50.0–81.3)	40.6 (25.0–62.5)	53.1 (39.1–62.5)	46.9 (35.9–62.5)	37.5 (28.1–53.1)
V. Monitoring and surveillance	75.0 (63.9–84.0)	75.0 (66.7–86.1)	70.8 (63.9–83.3)	81.9 (71.5–86.1)	80.6 (72.9–95.8)	58.3 (56.9–69.4)
VI. Internal communication	62.5 (48.4–75.0)	81.3 (56.3–93.8)	62.5 (45.3–75.0)	62.5 (50.0–75.0)	81.3 (64.1–90.6)	56.3 (43.8–62.5)
**Global**	**61.2** **(49.4–68.1)**	**78.4** **(59.0–85.0)**	**61.2** **(47.8–68.0)**	**63.1** **(52.8–68.8)**	**70.0** **(59.2–84.3)**	**54.4** **(39.7–59.8)**

**Table 2:** Part (**a**) Checklist results: Commitment of authorities and organization of the antimicrobial stewardship program (expressed in means); Part (**b**) Checklist results: Interventions aimed at optimizing the use of antimicrobials (expressed in means); Part (**c**) Checklist results: Education, monitoring, and internal communication (expressed in means).

(a)						
**Component**	Abbreviated Question	Fully Implemented, %	Partially Implemented, %	Planned but Not Initiated, %	Not Planned but Considered a Priority, %	Not Considered for Implementation, %
**I. Commitment** **of the authorities**	1a. Is the optimization of antimicrobials among the priorities of Senior Management?	23.1	38.8	29.8	5.0	3.3
	1b. Are ASP interventions included in annual plans, along with basic indicators?	22.3	30.6	28.9	13.2	5.0
	1c. Has Senior Management allocated financial and human resources to ASP?	19.8	33.1	22.3	13.2	11.6
	1d. Is there a formal document, approved by Management, for the implementation of ASP?	33.9	24.8	19.0	16.5	5.8
	2a. Is there an action plan or program in place that describes and prioritizes the interventions?	28.1	32.2	24.0	13.2	2.5
	2b. Is there a mechanism that monitors and measures the implementation of interventions?	20.7	30.6	32.2	10.7	5.8
	3a. Does the action plan on antimicrobial optimization receive specific funding?	9.9	12.4	12.4	28.9	36.4
	3b. Has an annual budget been drawn up for the implementation of ASP?	5.0	12.4	16.5	24.8	41.3
**II. Organization,** **structure, and accountability**	4.a Does the health facility have a multidisciplinary Committee on ASP with defined attributions?	38.0	26.4	24.0	8.3	3.3
	4b. Does the ASP Committee or another relevant committee meet regularly (at least quarterly)?	47.1	24.0	17.4	8.3	3.3
	5a. Does the health facility have a specialized ASP leader?	44.6	26.4	13.2	12.4	3.3
	5b. Has the team leader been assigned protected time for ASP in their job description?	28.1	24.0	24.8	14.0	9.1
	5c. Is there an epidemiologist and/or infectious disease specialist with protected time for ASP?	32.2	25.6	10.7	22.3	9.1
	5d. Is there a clinical pharmacist or pharmacist trained in infectious diseases with protected time for ASP?	15.7	27.3	14.9	26.4	15.7
	5e. Is there a microbiologist trained in the identification of AMR with protected time?	27.3	28.1	20.7	13.2	10.7
	6a. Does an ASP team exist with defined attributions?	33.1	25.6	24.8	13.2	3.3
	6b. Does the ASP team meet regularly?	25.6	23.1	28.9	17.4	5.0
	7. Do other health professionals outside ASP participate in optimization activities?	19.8	25.6	27.3	12.4	14.9
	8a. Does ASP collaborate with other healthcare teams in the facility (e.g., IPC)?	34.7	29.8	24.0	6.6	5.0
	8b. Does the ASP Team integrate and coordinate its activities with members of other teams (e.g., IPC)?	38.8	26.4	19.0	12.4	3.3
	8c. Does the ASP team have an assigned office or physical space for work and meetings?	32.2	11.6	10.7	20.7	24.8
	8d. Does the health facility have its own microbiology laboratory, or use an external one?	86.8	9.9	0.8	1.7	0.8
	8e. Does the microbiology laboratory collect and process samples seven days a week?	75.2	19.8	2.5	1.7	0.8
	8f. Is access to laboratory results available through medical records or patient charts?	89.3	8.3	1.7	0.8	0.0
	8g. Does the microbiology laboratory report cultures with sensitivity testing?	92.6	6.6	0.0	0.8	0.0
	8h. Does the microbiology laboratory generate selective sensitivity reports?	57.0	20.7	9.1	5.0	8.3
	8h. Does the microbiology laboratory have rapid diagnostic techniques?	42.1	17.4	13.2	15.7	11.6
	9a. Does ASP periodically issue descriptive reports of program activities?	21.5	19.0	45.5	9.1	5.0
	9b. Is the intervention report disseminated to Management, care teams, and national authorities?	26.4	19.0	38.8	12.4	3.3
(**b**)						
**Component**	**Abbreviated Question**	**Fully Implemented,** **%**	**Partially Implemented,** **%**	**Planned but Not Initiated, %**	**Not Planned but Considered a Priority, %**	**Not Considered for Implementation, %**
**III. Interventions aimed at** **optimizing the use of antimicrobials**	10a. Does the health facility have a standard antimicrobial therapeutic guideline?	39.7	27.3	21.5	8.3	3.3
	10b. Are antimicrobial treatment guidelines reviewed and updated periodically?	34.7	29.8	20.7	10.7	4.1
	10c. Do the clinical practice guidelines (CPGs) include empirical treatment, dosage, and duration, oral options, among others?	36.4	28.9	21.5	10.7	2.5
	10d. Does the facility have CPGs for surgical prophylaxis, updated in the last 5 years?	52.9	24.8	14.0	7.4	0.8
	10e. Does the facility have CPGs for CAP, updated at least once in the last 5 years?	43.0	25.6	19.0	9.1	3.3
	10f. Does the facility have CPGs for HAP (including VAP), updated in the last 5 years?	37.2	29.8	21.5	9.1	2.5
	10g. Does the facility have CPGs for UTI, updated at least once in the last 5 years?	47.1	25.6	15.7	9.1	2.5
	10h. Does the facility have CPGs for skin and soft tissue infections, updated?	35.5	23.1	26.4	10.7	4.1
	10i. Does the facility have CPGs for IA infections updated in the last 5 years?	29.8	22.3	28.9	13.2	5.8
	10.j Does the facility have CPGs for bacteremia/sepsis, updated in the last 5 years?	34.7	26.4	23.1	12.4	3.3
	10k. Does the facility have CPGs for medical prophylaxis and infections in immunocompromised patients?	28.1	23.1	24.8	16.5	7.4
	10l. Does it have CPGs for the treatment of MDR infections, updated in the last 5 years?	20.7	28.1	28.1	15.7	7.4
	11. Is a review or audit of antibiotic therapy carried out periodically?	25.6	26.4	27.3	16.5	4.1
	12. Do prescribers have easy access to ASP advice and feedback?	35.5	23.1	20.7	17.4	3.3
	13. Do members of the ASP team regularly conduct ward rounds?	33.1	29.8	22.3	12.4	2.5
	14a. Does the health facility have a list of authorized antimicrobials?	54.5	23.1	10.7	7.4	4.1
	14b. Does the pharmacopeia include a list of restricted or controlled ASP?	38.8	24.0	20.7	10.7	5.8
	14c. Does at least one team member participate in the selection of antimicrobials?	43.8	22.3	13.2	14.9	5.8
	15. Does the health facility have access to laboratory and imaging diagnostic services?	76.0	16.5	4.1	2.5	0.8
	16. Are there IT services, control sheets, etc., that can be used to collect data?	49.6	29.8	7.4	9.1	4.1
	17a. Are there harmonized support documents for treatment and antimicrobial optimization?	63.6	19.8	8.3	6.6	1.7
	17b. Is there a written policy requiring complete documentation of antimicrobial indications?	37.2	24.8	17.4	14.0	6.6
	17c. Is there an ASP team member with broad availability for consultations?	30.6	24.0	16.5	19.0	9.9
	17d. Are antimicrobial prescriptions reviewed and does ASP provide suggestions within 48–72 hours of initiation?	38.8	34.7	15.7	9.9	0.8
	17e. Is dispensing of antimicrobials by pharmacy interrupted (“auto-stop”)?	9.1	20.7	28.9	25.6	15.7
	17f. Is antimicrobial therapy promoted to switch from IV to oral in appropriate situations?	39.7	35.5	14.9	7.4	2.5
	17g. Is dose optimization carried out using pharmacokinetics and pharmacodynamics?	43.8	32.2	15.7	7.4	0.8
	17h. Have strategies been developed for AM dose adjustments in cases of organ dysfunction?	17.4	30.6	23.1	19.8	9.1
	17i. Are there automatic alerts when treatment may be unnecessarily duplicated?	11.6	16.5	24.0	28.1	19.8
	17j. Does the hospital have guidelines for therapeutic drug monitoring (e.g., vancomycin)?	19.0	19.8	19.8	23.1	18.2
	17k. Are there alerts for the use of aminoglycosides in once-daily dosing?	10.7	13.2	26.4	24.8	24.8
(**c**)						
**Component**	**Abbreviated Question**	**Fully Implemented,** **%**	**Partially Implemented,** **%**	**Planned but Not Initiated, %**	**Not Planned but Considered a Priority, %**	**Not Considered for Implementation, %**
**IV. Education and training**	18. Does the health facility include ASP topics in the initial training of its staff?	9.9	26.4	28.1	24.8	10.7
	19. Does it offer its staff continuous education or professional development in ASP and IPC?	17.4	40.5	15.7	20.7	5.8
	20a.Does the facility train the ASP team in this field and in IPC?	20.7	32.2	14.9	19.8	12.4
	20b. Does the facility offer education/information on ASP to patients and their families?	11.6	23.1	19.8	24.0	21.5
**V. Monitoring and surveillance**	21. Does the ASP Committee or relevant team conduct audits or point prevalence surveys?	28.9	28.1	19.8	17.4	5.8
	22a. Does the facility periodically monitor the acquisition, prescription, and dispensing of ASP?	40.5	24.8	21.5	12.4	0.8
	22b. Does the facility monitor problems of shortage or lack of essential ASP?	52.9	29.8	9.9	5.8	1.7
	22c. Does it have a mechanism for reporting defective and falsified medicines?	38.8	32.2	9.1	12.4	7.4
	22d. Is antimicrobial consumption categorized according to the WHO AWaRe classification?	18.2	17.4	26.4	20.7	17.4
	23. Does ASP periodically monitor antibiograms of a basic set of indicator bacteria?	47.1	26.4	21.5	5.0	0.0
	24a. Does the ASP team monitor compliance with at least one specific intervention?	39.7	26.4	20.7	10.7	2.5
	24b. Does the IPC committee monitor infection rates from MDR microorganisms?	72.7	22.3	5.0	0.0	0.0
	24c. Does the Infection Prevention and Control Committee monitor *C. difficile* infection rates?	52.1	24.8	12.4	7.4	3.3
**VI. Internal communication** **in the health facility**	25. Does it analyze and report to prescribers and Management the antimicrobials acquired, prescribed, and dispensed?	28.1	23.1	31.4	13.2	4.1
	26. Does it review, analyze, and report sensitivity rates and share the results with prescribers?	33.9	26.4	28.9	10.7	0.0
	27. Does it communicate audit results to prescribers, along with concrete action points?	17.4	25.6	38.8	14.9	3.3
	28. Does the facility produce an integrated sensitivity report and update it periodically?	42.1	24.0	20.7	13.2	0.0

**Table 3:** SWOT analysis of antimicrobial stewardship programs implementation.

Strengths	Weaknesses
ASP frequently fromalized within hospital structures. High commitment from core ASP multidisciplinary teams. Core interventions (CPG, audits, joint rounds) are already operational. Access to CML. Increasing involvement of clinical pharmacy services.	Limited support from hospital authorities and limited protected time for leaders and/or pharmacists. Scarcity of pharmacists involved in the ASP. Underutilization of CML diagnostic capacity. Overuse of critical antimicrobials (e.g., ceftriaxone, carbapenems, and vancomycin). Suboptimal adherence to surgical prophylaxis guidelines. Interventions conducted without a clearly defined strategy or subsequent assessments of their effectiveness. Weak internal communication (e.g., type of interventions, dissemination of CPG, results of surveillance and monitoring) to hospital authorities and prescribers.
**Opportunities**	**Threats**
Formalize the ASP organization in a document. Reinforce ASP team building. Obtain protected time for team leaders/pharmacists. Reduce the use of ceftriaxone and closely monitor prescriptions of carbapenems and vancomycin. Share results of the PPS and the checklist with the hospital’s administrative leaders and prescribers to increase support to the program. Assess compliance with CPG (e.g., surgical prophylaxis) Production and dissemination of cumulative antibiograms for prevalent bacterial groups (e.g., GNB).	Selection of AMR due to critical drug overuse. Exhaustion of the team due to both resources constrains and lack of protected time. Unsustainability of the program due to a poor formalization and funding.

Abbreviations: ASP: antimicrobial stewardship programs; CML: clinical microbiology laboratories; CPG: clinical practice guidelines. GNB: Gram-Negative Bacilli; AMR: antimicrobial resistance.

**Table 4:** Main strategic recommendations for improving hospital Antimicrobial Stewardship Programs.

Intervention Area	Strategic Recommendations
**Administration and organization of the ASP**	Formalize the ASP and the team in a document outlining its place within the hospital’s structure and including a program description, essential team members (physician, pharmacist, microbiologist, and infection control staff), and their responsibilities. Ensure the integration of clinical pharmacists—where they are not already involved—and allocate enough time for them and program leaders.
**Strategic action planning**	Design a strategic action plan prioritizing three to four key activities during the first year, generally including control of ceftriaxone, carbapenems, and vancomycin use; production of updated CPG jointly with prescribers; and monitoring of ASP interventions, antibiotic consumption, and AMR.
**Diagnostic optimization**	Optimize the use of the CML: increase sample collection before prescribing antimicrobials; periodically produce cumulative antibiograms; incorporate Nucleic Acid Amplification Test technology when possible; and enhance communication between CML, prescribers, and the ASP team.
**Multimodal education**	Ensure that educational initiatives are strategically aligned with the action plan objectives, implemented through multimodal approaches (e.g., incidental—during joint clinical rounds or pre-authorization of antibiotics structured—through courses or conferences).

ASP: antimicrobial stewardship programs; CML: clinical microbiology laboratories; CPG: clinical practice guidelines.

## Data Availability

Data are unavailable due to privacy and ethical restrictions. Please direct any further inquiries to the corresponding author.
